# *Atahualpacorishenryi*, a new species of plant bug from Colombia (Heteroptera, Miridae, Mirini)

**DOI:** 10.3897/zookeys.796.20801

**Published:** 2018-11-15

**Authors:** Paulo Sérgio Fiuza erreira, Jose Luis Benavides Lopes, Fagner de Souza, Luciano Santana Fiuza erreira

**Affiliations:** 1 Universidade Federal de Viçosa, Departamento de Entomologia, Viçosa, MG, Brazil Universidade Federal de Viçosa Viçosa Brazil; 2 Muséum National d´Histoire Naturelle, Département Systématique et Evolution, Paris, France Muséum National d´Histoire Naturelle Paris France; 3 Universidade Federal do Triângulo Mineiro, Laboratório de Ecologia Aquática, Avenida Guilherme Ferreira, 1940, Uberaba, Minas Gerais, Brazil Universidade Federal do Triângulo Mineiro Uberaba Brazil; 4 Universidade de Viçosa, Faculdade de Ciências Biológicas e da Saúde, Engenharia Ambiental. Av. Maria de Paula Santana, nº 3815- Bairro Silvestre Viçosa, Minas Gerais, 36570-000, Brazil Universidade de Viçosa Viçosa Brazil

**Keywords:** *
Atahualpacoris
*, description, morphology, Neotropical Miridae

## Abstract

A new species of plant bug in the genus *Atahualpacoris* Carvalho, tribe Mirini, is described. Morphological characters differentiating *Atahualpacoris* from the related genus *Calocorisca* are provided, and a diagnosis of each known species of *Atahualpacoris* is presented. Specimens of the new species were collected by light trap in a natural ecosystem of the Andes Mountains, Department of Tolima, Colombia. The adult and male genitalia are illustrated.

## Introduction

Carvalho (1985) described *Atahualpacoris* in the subfamily Mirinae, tribe Mirini. Currently, the genus contains six species distributed in Andean America: *A.columbiensis* Carvalho, 1985 (Colombia and Peru), *A.impunctatus* Carvalho, 1985 (Venezuela), *A.incaicus* Carvalho, 1985 (Peru), *A.lojaensis* Carvalho, 1985 (Ecuador), *A.tamboensis* Carvalho, 1985 (Colombia, Ecuador and Peru), and *A.venezuelensis* Carvalho, 1985 (Venezuela) (Carvalho 1985, Schuh 2002–2013).

In this paper, a new species, *Atahualpacorishenryi*, is described. Illustrations of the adult and male genitalia and a key to species are provided to facilitate species recognition.

## Materials and methods

Specimens were collected by light trap in the central cordillera of the Andes mountains, Cajamarca, at Tolima department of Colombia. The collection areas correspond to Veredas: Cristales La Paloma, Vereda La Luisa and include Montane Wet Forest and Very Humid Montano Low Forest, with elevations ranging from 2100 to 3200 meters above sea level. Rainfall exceeds 2000 mm/year, and temperatures are lower than 18 °C. The rugged topography is characterized by steep slopes (Holdridge 1967).

Ten traps were used to take 11 samples in two areas from May 2012 to March 2013. Traps (Luiz de Queiroz model) with a UV 15-watt fluorescent lamp were installed 2 m above ground (Ferreira and Martins 1982). Traps were activated one day a week from 1800–0700 hr and all specimens were collected after 0700 hr.

Male genitalia were prepared by immersing them in room temperature KOH for 24 hours until they were softened and cleared. After being rinsed in distilled water, genitalia were placed in an excavated microscopic slide containing glycerol, and the endosoma, right and left parameres, and phallotheca were dissected using a Leica M205 A stereoscope. Material was preserved in glycerin in a microvial that was the pinned below the specimen. Terminology for male genitalia follows Cassis (2008). Images of the adult male and male genitalia were captured using a Leica MC170 HD digital microscope camera.

The holotype and paratypes are deposited in the Regional Museum of Entomology of the Federal University of Viçosa, MG, Brazil (UFVB).

## Taxonomy

### Atahualpacoris

Taxon classificationAnimaliaHemipteraMiridae

Carvalho

#### Diagnosis.

Medium to large size (6–10 mm), oval-elongate, covered with short, adpressed pubescence. Front striated and vertex grooved. Pronotum trapeziform, narrowed to head, strongly punctate with lateral margins rounded; calli smooth; collar shiny and well defined. Scutellum rough and punctate, shiny and swollen. Hemelytra smooth, covered with short, adpressed pubescence.

#### Remarks.

*Atahualpacoris* and *Calocorisca* (Carvalho 1985, 1986) are morphologically similar genera. *Atahualpacoris* has the body elongate with the lateral margins parallel or nearly so, and the scutellum is convex and raised at the middle, with its apex acute, slightly curled upward. The hemelytral membrane length is longer than in *Calocorisca* (*Atahualpacoris* length of hemelytra 1.7 to 1.9 times length of membrane; *Calocorisca* length of hemelytra 2.2 to 2.5 times length of membrane). Antennal segment I is covered by short setae mixed with common hairs, whereas in *Calocorisca* this segment is covered by erect setae longer than the width of the segment.

##### Key to the species of *Atahualpacoris*

**Table:** 

1	General color brown or dark brown to black	**2**
–	General color light brown, yellowish brown or reddish brown	**6**
2	Collar of pronotum brown to dark brown; antenna uniformly brown or yellowish, or apex of segments II, III and IV, black; scutellum uniformly brown or dark brown with a pale longitudinal stripe or with yellowish spots. Male: endosoma with one or two spicules; longer spicule lacking many flat spines on ventral surface; area near secondary gonopore with spines, denticles or serrate edges; left paramere with sensorial lobe not crenulate	**3**
–	Collar yellowish; scutellum not uniformly brown, with two lateral irregular stripes and apex yellow; antennal segment I yellowish with a basal and lateral black spots; II to IV black with apex yellow. Male: endosoma with three spicules; area near secondary gonopore absent; left paramere C- shaped and twisted toward broad apex; sensorial lobe stout and weakly crenulate	***A.henry* sp. n.**
3	Clavus brown; hemelytron membrane darkened; scutellum dark brown without pale longitudinal stripe. Male: endosoma with one spicule; area near secondary gonopore with outer margin bearing long spine, smaller spines of different sizes and denticles, or with enlarged apical region with edge serrate, or broad area with dorsal margin formed by series of denticles and one acute elongate extension	**4**
–	Clavus with longitudinal yellowish stripe following claval vein; hemelytron membrane pale with numerous small brown spots; scutellum dark brown with pale yellowish spots, apex and longitudinal median stripe pale. Male: endosoma with two spicules, a longer spicule curved, apex acute, a small spicule more robust, thickened to tip with apex curved and sharp; area near secondary gonopore with distal region denticulate; left paramere with well-developed sensorial lobe nearly 1/3 of paramere length, apical region twisted, apex broad with lateral edges acute	***A.columbiensis* Carvalho**
4	Scutellum and cuneus uniformly brown; hemelytron membrane with two light spots on outer margin, and veins pale. Male: endosoma with long, narrow and curved spicule tapered in apical 1/3, with apex acute; area near secondary gonopore with outer margin bearing long spine, smaller spines of different sizes, and denticles; left paramere with sensorial lobe bearing small tubercles, apex forked and twisted	***A.impunctatus* Carvalho**
–	Not as above	**5**
5	Antenna yellowish; scutellum dark brown with apex and scattered spots pale; cuneus with small pale spots; hemelytron membrane darkened with many small brown spots. Male: endosoma with long spicule, apical third tapered and curved; area near secondary gonopore with enlarged apical region with serrated edge; left paramere strongly curved; sensorial lobule well developed, very small denticles on surface	***A.incaicus* Carvalho**
–	Antenna brown; scutellum dark brown with small spots at apex yellowish; cuneus with small rounded yellowish spots; hemelytron membrane darkened with numerous small yellowish spots. Male: endosoma with long, broad spicule, apex tapering and curved; area near secondary gonopore broad, its dorsal margin formed by series of denticles and acute, elongate spinelike extension; left paramere sickle-shaped with apex blunt; sensorial lobe small, rounded, dorsal surface with many small denticles and long hairs	***A.venezuelensis* Carvalho**
6	Antenna yellowish; pronotum yellowish with brown spots; collar black; scutellum dark brown with apex and small spots whitish; clavus and cuneus with small black spots along claval suture; hemelytron membrane darkened with scattered small brown spots, and large pale spot close to apex of cuneus; Male: endosoma with long stout spicule, apical 1/3 bent at right angle, extreme apex tapered and twisted; area near secondary gonopore absent; left paramere with sensorial lobe developed and flattened; apex strongly curved and blunt	***A.jojaensis* Carvalho**
–	Antennal segment I reddish with tiny sclerotized black dots; pronotum reddish with numerous small yellowish spots; collar reddish; scutellum with brown and yellowish spots; clavus reddish brown; cuneus with basal area dark; hemelytron membrane darkened with many small brown spots and red veins. Male: endosoma with long spicule, curved and tapering to apex; area near secondary gonopore with apical margin serrate; left paramere falciform, sensorial lobe strongly developed with long hairs, apex twisted and blunt	***A.tamboensis* Carvalho**

### Atahualpacoris henryi gen. n.

Taxon classificationAnimaliaHemipteraMiridae

http://zoobank.org/D976195C-C98A-414F-A631-44087BAE7601

Figs 1 , 2 , 3 , 4

#### Diagnosis.

Distinguished from other species of *Atahualpacoris* by large size (8.0 to 9.5 mm) with general color dark brown to black; hemelytra dark brown; two large yellowish spots on corium; cuneus with yellowish spots near cuneal fracture on inner basal angle and at apex; hemelytral membrane darkened with many small, rounded, scattered, pale spots; absence of black spot at base of larger cells of hemelytral membrane; male genitalia: endosoma with three spicules of different size and shape, acute distally; longer spicule with many flat spines on ventral surface; left paramere sickle shaped, apex broad and twisted, sensorial lobe crenulate.

**Figure 1.**
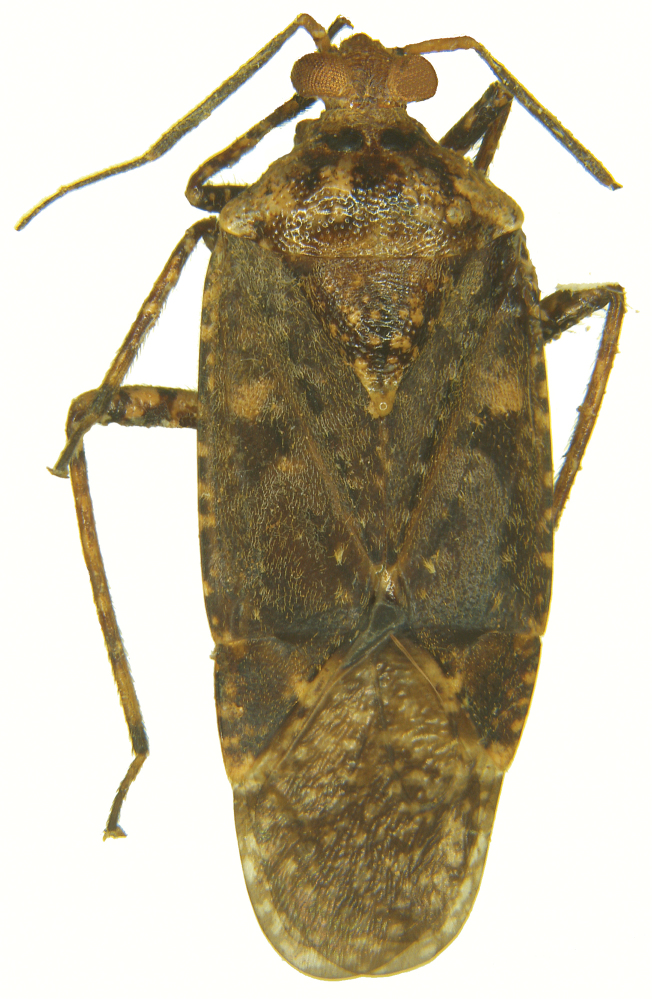
*Atahualpacorishenryi* sp. n. male, holotype (dorsal view).

**Figure 2.**
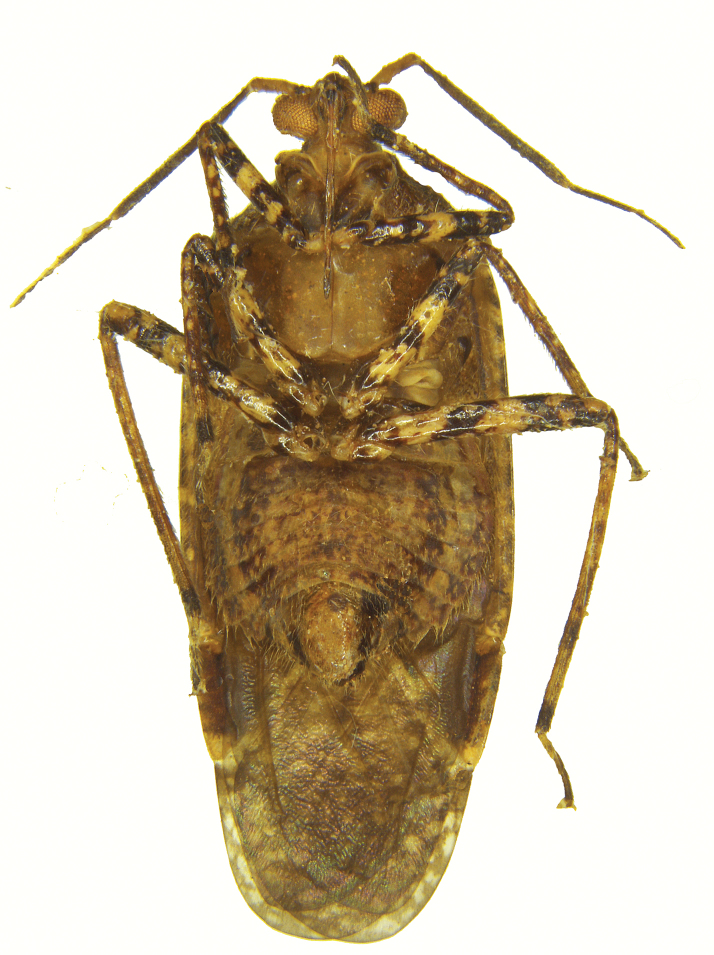
*Atahualpacorishenryi* sp. n. male, holotype (ventral view).

**Figure 3.**
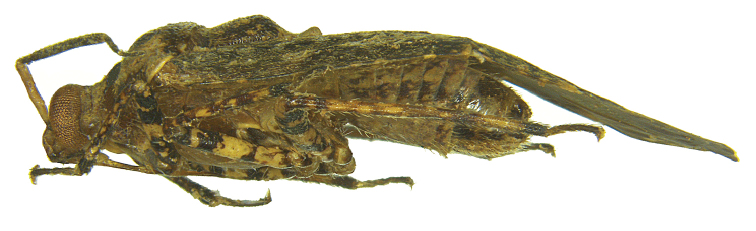
*Atahualpacorishenryi* sp. n. male, holotype (lateral view).

Holotype male (Figs 1–3); measurements (Table 1).

**Table 1.** Characters measurements in millimeters taken from Holotype first, followed by range (minimum and maximum) of five males and five females specimens, and average for each character.

Caracteres	Holotype	Male			Female		
		min	max	average	min	max	average
Body length	9	7,60	9,00	8,60	8,40	10,00	9,44
Body width	3,5	3,10	3,70	3,40	3,40	3,80	3,60
Head length	0,7	0,50	0,70	0,62	0,50	0,70	0,60
Head width	1,4	1,30	1,40	1,32	1,30	1,40	1,34
Head distance between eyes	0,36	0,33	0,38	0,36	0,44	0,49	0,46
Antennal segment I length	0,90	0,79	0,90	0,85	0,79	0,87	0,84
Antennal segment II length	1,97	1,90	2,41	2,09	1,87	2,10	2,03
Antennal segment III length	0,79	0,79	0,92	0,86	0,82	0,92	0,88
Antennal segment IV length	0,59	0,59	0,69	0,65	0,62	0,67	0,64
Pronotum length	1,38	1,21	1,41	1,35	1,18	1,38	1,32
Pronotum width at base	3,05	2,72	3,08	2,95	3,03	3,25	3,16
Hind femur length	2,38	2,21	2,64	2,45	0,85	0,92	0,89
Hind tibia length	3,31	3,16	3,50	3,33	3,22	3,47	3,33
Hind tarsus length	0,90	0,79	0,92	0,88	0,85	0,92	0,89
Scutellum length	1,46	1,33	1,49	1,41	1,54	1,69	1,59
Scutellum width	1,54	1,36	1,62	1,49	1,59	1,69	1,64
Hemelytron length	7,27	6,45	7,60	7,16	6,94	8,43	7,95
Cuneus length	1,41	1,05	1,41	1,30	1,41	1,64	1,53
Cuneus width	1,33	1,18	1,46	1,35	1,26	1,49	1,41
Rostrum length	2,22	2,13	2,75	2,41	2,19	2,81	2,39

**Table 2.** Results of Principal Component Analysis for *Atahualpacorishenryi* sp. n. (PCA). Bold values are the ones that most influence the divergences.

**Jolliffe cut-off: 0,003**		
	**PCA1**	**PCA2**
**Eigenvalue**	0,073315	0,005824
% **Variance**	83,874	6,6633
**Body length**	0,003118	0,02205
Body width	0,03958	0,2028
Head length	**0,1118**	0,1741
Head width	0,07589	0,2386
Head distance between eyes	-**0,1176**	0,3677
Antennal segment I	**0,1056**	0,2467
Antennal segment II	**0,1111**	0,1195
Antennal segment III	0,06692	0,2308
Antennal segment IV	**0,1068**	0,2588
Pronotum length	**0,1088**	0,213
Pronotum width at base	0,0297	0,2007
Hind femur length	**0,9408**	-0,1782
Hind tibia length	0,08981	0,2269
Hind tarsus length	0,07514	0,1453
Scutellum length	-0,01467	0,257
Scutellum width	0,006607	0,2507
Hemelytron length	-0,00684	0,005071
Cuneus length	-**0,05448**	0,1889
Cuneus width	0,06587	0,4241
Rostrum length	0,08813	0,07838

**Table 3.** Comparison of morphometric characters (in percentage) used in the diagnosis of sexual dimorphism of *Atahualpacorishenryi* sp. n. Only characters with greater percentage differences of the relations between the characters. Min. = Minimum values. Max. = Maximum values. DP = standard deviation.

CHARACTERS	Holotype (male)	Male				Female			
		Min.	Max.	Average	DP	Min.	Max.	Average	DP
**Body length**									
Hind femur length	26,50	26,50	30,71	**28,54**	1,64	8,81	10,07	**9,42**	0,47
**Body width**									
Hind femur length	68,13	68,13	77,68	**72,15**	3,71	23,56	25,64	**24,67**	1,03
**Head length**									
Hind femur length	29,35	22,67	29,35	**25,33**	3,40	54,17	80,29	**67,82**	9,40
**Head width**									
Head distance between eyes	25,64	25,64	29,59	**27,22**	1,65	31,14	35,50	**34,09**	1,84
Hind femur length	170,33	169,63	203,16	**185,94**	15,69	64,10	69,03	**66,24**	1,90
**Head distance between eyes**									
Antennal segment I	40,00	37,14	45,45	**42,30**	3,61	51,52	56,25	**54,61**	1,87
Hind femur length	15,05	12,62	16,28	**14,71**	1,32	48,57	54,55	**51,46**	2,33
Hind tarsus length	40,00	37,14	45,16	**41,06**	3,44	48,57	54,55	**51,46**	2,33
**Hind femur length**									
Antennal segment I	37,63	32,63	37,63	**34,83**	2,44	88,57	97,06	**94,27**	3,45
Antennal segment II	120,78	107,45	127,16	**117,78**	7,18	41,46	45,21	**43,83**	1,42
Pronotum length	172,22	172,22	194,34	**181,86**	9,49	62,96	76,09	**67,56**	5,31
Pronotum width at base	78,15	78,15	88,79	**82,97**	3,94	27,35	28,43	**28,05**	0,44
Leg hind tibia length	71,99	69,87	77,53	**73,52**	2,85	25,13	27,61	**26,65**	0,99
Leg hind tarsus length	265,71	263,89	297,06	**279,67**	15,53	100,00	100,00	**100,00**	0,00
Scutellum length	163,16	163,16	190,57	**174,52**	10,97	54,55	57,38	**55,83**	1,17
Scutellum width	155,00	155,00	174,58	**164,98**	8,38	51,52	56,45	**54,09**	2,16
Hemelytron length	32,79	32,04	37,60	**34,29**	2,15	10,66	12,19	**11,19**	0,59
Cuneal length	169,09	169,09	209,76	**189,43**	16,30	53,13	63,64	**58,01**	3,90
Cuneal width	178,85	177,19	186,96	**181,23**	4,06	56,90	71,43	**63,51**	6,40
Rostrum length	107,48	91,70	115,77	**102,58**	9,85	31,00	39,89	**37,35**	3,60
**Cuneal length**									
Antennal segment I	63,64	59,62	78,05	**65,99**	7,66	51,56	56,36	**54,58**	1,87
Pronotum length	98,18	98,18	114,63	**104,14**	6,78	83,64	88,52	**85,93**	2,24
Leg hind femur length	169,09	169,09	209,76	**189,43**	16,30	53,13	63,64	**58,01**	3,90
Leg hind tibia length	42,57	33,31	42,57	**39,04**	3,53	42,98	47,34	**46,03**	1,86

#### Description.

*Body* shiny, parallel sided, length ca. 2.5 times width, dorsal vestiture with short, semi-erect hairs; general color dark brown to black with spots and small marks yellowish. **Head** declivous, shiny, broader than long, weakly convex dorsally; in dorsal view dark brown with yellowish stripes; frons with longitudinal median groove; eyes prominent, contiguous to collar, occupying most of head in dorsal view; vertex with transverse sulcus; antennal vestiture with short pilosity, less than width of segment; antennal fossa above jugal-loral suture dark brown; antennal segment I shorter than width of vertex between eyes, bearing short setae mixed with common hairs, yellowish with extreme base and lateral spots black; segments II–IV black, extreme apex yellow, with whitish adpressed pilosity; relative lengths of antennal segments in ascending order II < I < III< IV; eyes black; rostrum brown with light brown spots, reaching mesosternum; lateral margins of clypeus, posterior area of juga and loral-jugal suture dark brown; epipharynx brown at base. **Pronotum** dark brown, trapeziform, two times wider than long, vestiture with sparse, short, pale hairs; collar yellowish; callus black, swollen, shiny, smooth with lateral sides slightly marginate; disc of pronotum convex, shiny, distinctly rugose and punctate with irregular yellowish spots; humeral angles slightly depressed; pronotum ventrally with xypho of prosternum yellowish with brown spots; propleura dark brown, shiny, rugose and punctate with sparse, adpressed hairs; mesosternum dark brown, slightly rugose and punctate; mesepisternum and metaepisternum dark brown; ostiolar peritreme brown with median lobe developed and evaporative area yellowish; coxae and femora with semierect hairs shorter than width of segment; coxae black with yellowish spots; posterior margins of median coxae yellowish; femora black with scattered yellowish spots; tibiae brown with scattered yellowish spots, parallel rows of tiny black spines along entire length, and vestiture of common semi-adpressed pale hairs mixed with dark bristles shorter or equal to width of segment; length of hind tibia more than 3.5 times length of hind tarsus; tarsi brown, darkening to apex. **Scutellum** triangular, rugose, shiny, slightly convex, and raised at middle, with apex acute, slightly curled upward; general color dark brown with two lateral irregular stripes, apex yellow; clothed with sparse semi-erect hairs. **Hemelytron** dark brown, elongate, subparallel, lateral margins slightly rugose with short, pale, adpressed pubescence mixed with dark hairs; clavus black; two large, somewhat rounded spots on corium, and small patches throughout embolium yellowish; claval-corial suture and embolio-corial suture impunctate; embolium delimited along entire length; cuneus slightly longer than wide with small spots on outer margin, broad spot near cuneal fracture, spot on inner basal angle and apex yellowish; hemelytral membrane opaque, glabrous and slightly rugose, darkened with many scattered, small, pale spots. **Abdomen** brown with yellowish areas, vestiture with adpressed or semi-erect hairs.

**Figure 4.**
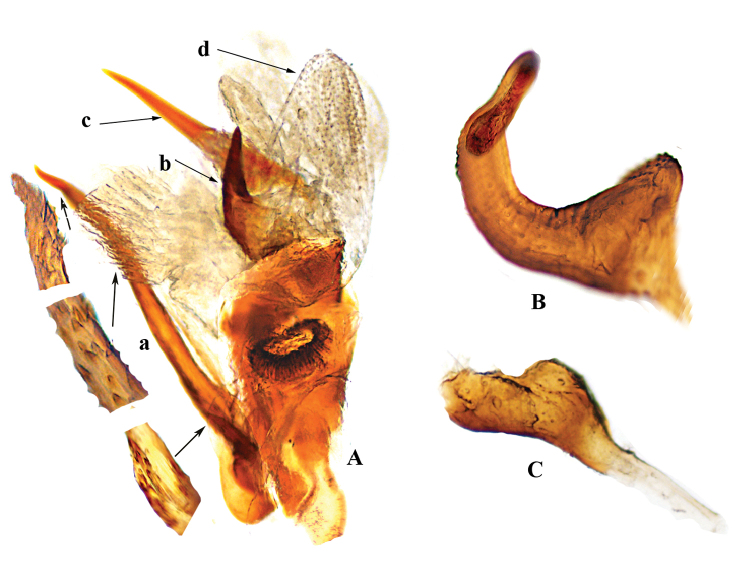
*Atahualpacorishenryi* sp. n. male, holotype **A** endosoma **a** longer spicule with many flat spines **b** shorter sickle-shaped spicule **c** third spicule larger at base **d** membranous lobes with tiny teeth **B** left paramere **C** right paramere.

**Male genitalia** (Figure 4). Endosoma (Figure 4A) with membranous lobes covered with tiny teeth (Figure 4Ad), and three thick spicules of different size and shape, acute distally; longer process narrow, rising below secondary gonopore, ventral surface with many flat spines (Figure 4Aa); second process shorter, broad, sickle-shaped (Figure 4Ad); third process larger at base, narrowed to acute apex (Figure 4Ac); secondary gonopore large, distinct, with rim wide and ribbed. Left paramere (Figure 4B) C-shaped in dorsal aspect, twisted toward broad apex; sensorial lobe relatively stout and weakly crenulate. Right paramere (Figure 4C) reduced, sensorial lobe relatively stout.

**Female.** Similar to males in structure and vestiture. Measurements (Table 1).

#### Geographic distribution.

Colombia (Tolima).

#### Host plant.

Unknown.

#### Etymology.

Named in honor of Dr. Thomas J. Henry (National Museum of Natural History, Washington D.C.) for his great contributions to the knowledge of Heteroptera, especially the family Miridae.

#### Material examined.

**Holotype** male, Colombia,Tolima, Cajamarca, La Colosa, C. Andina, Armadilha Luminosa, Febrero 2013, Benevides Lopes J.L.(ICN- Instituto de Ciencias Naturales, Universidad Nacional de Colombia, Bogotá) **Paratypes**: (Same locality of holotype): female, IX/2012; male, 4 females, XI/2012; 3 males, female, XII/2012; male, 2 females I/ 2013; 4 males, female II/ 2013 (UFVB- Universidade Federal de Viçosa, MG, Brazil).

## Supplementary Material

XML Treatment for Atahualpacoris

XML Treatment for Atahualpacoris henryi
